# Supplementary material for: AtRAC7/ROP9 Small GTPase Regulates A. thaliana Immune Systems in Response to B. cinerea Infection
Source: Int J Mol Sci. 2024 Jan 2;25(1):591. doi: 10.3390/ijms25010591 (PMC10779071; doi:10.3390/ijms25010591)
Supplement: Supplementary file 1 [file ijms-25-00591-s001.zip › Figure S1.pdf]

A

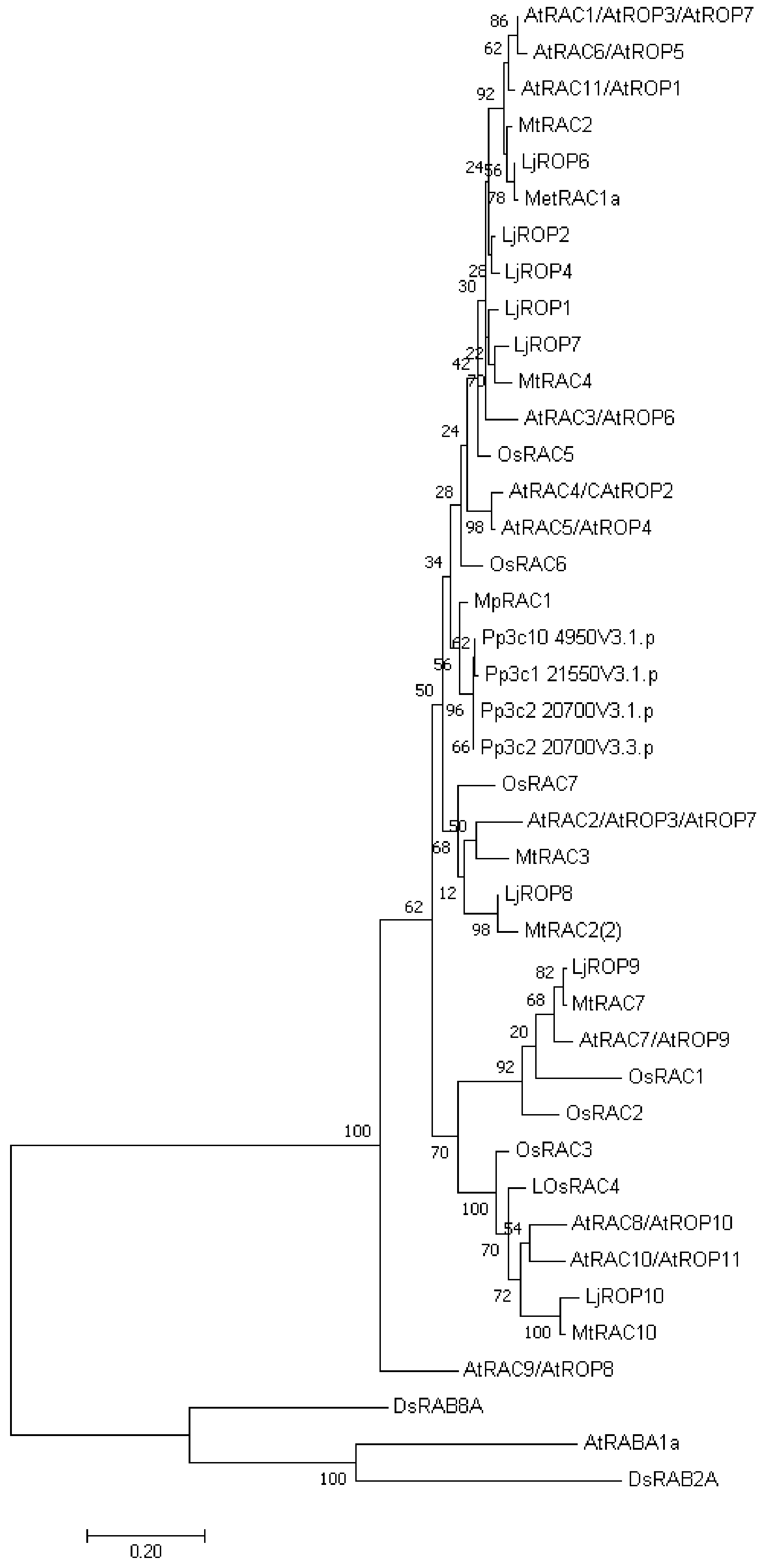

AtRAC1/AtROP3/AtROP7
AtRAC6/AtROP5
AtRAC11/AtROP1
MtRAC2
LjROP6
MetRAC1a
LjROP2
LjROP4
LjROP1
LjROP7
MtRAC4
AtRAC3/AtROP6
OsRAC5
AtRAC4/CAtROP2
AtRAC5/AtROP4
OsRAC6
MpRAC1
Pp3c10 4950V3.1.p
Pp3c1 21550V3.1.p
Pp3c2 20700V3.1.p
Pp3c2 20700V3.3.p
OsRAC7
AtRAC2/AtROP3/AtROP7
MtRAC3
LjROP8
MtRAC2(2)
LjROP9
MtRAC7
AtRAC7/AtROP9
OsRAC1
OsRAC2
OsRAC3
LOsRAC4
AtRAC8/AtROP10
AtRAC10/AtROP11
LjROP10
MtRAC10
AtRAC9/AtROP8
DsRAB8A
AtRABA1a
DsRAB2A
0.20

B

```
AtRAC1_ROP3_ROP    1 ------------MSASRFIKCVTVGDGAVGKTCLLISYTSNTFPTDYVPTVFDNFSANVV
AtRAC2_ROP3_ROP    1 ------------MSTARFIKCVTVGDGAVGKTCMLISYTSNTFPTDYVPTVFDNFSANVV
AtRac3_ROP6        1 ------------MSASRFIKCVTVGDGAVGKTCLLISYTSNTFPTDYVPTVFDNFSANVI
ATRAC4_ROP2        1 -------------MASRFIKCVTVGDGAVGKTCMLISYTSNTFPTDYVPTVFDNFSANVV
ATRAC5_ROP4        1 ------------MSASRFIKCVTVGDGAVGKTCMLISYTSNTFPTDYVPTVFDNFSANVV
ATRAC6_ROP5        1 ------------MSASRFIKCVTVGDGAVGKTCLLISYTSNTFPTDYVPTVFDNFSANVV
ATRAC7_ROP9        1 ------------MSASKFIKCVTVGDGAVGKTCMLICYTSNKFPTDYIPTVFDNFSANVA
ATRAC8_ROP10       1 ----------MASSASKFIKCVTVGDGAVGKTCMLICYTSNKFPTDYIPTVFDNFSVNVV
ATRAC9_ROP8        1 MSASMAATSTSSATATTFIKCVTVGDGAVGKTCLLISYTSNTFPTDYVPTVFDNFNANVL
ATRAC10_ROP11      1 ----------MASSASKFIKCVTVGDGAVGKTCMLICYTSNKFPTDYIPTVFDNFSANVV
ATRAC11_ROP1       1 ------------MSASRFVKCVTVGDGAVGKTCLLISYTSNTFPTDYVPTVFDNFSANVV

AtRAC1_ROP3_ROP   49 VNGATVNLGLWDTAGQEDYNRLRPLSYRGADVFILAFSLISKASYENVSKKWIPELKHYA
AtRAC2_ROP3_ROP   49 VDGSTVNLGLWDTAGQEDYNRLRPLSYRGADVFLLAFSLISKASYENIHKKWLPELKHYA
AtRac3_ROP6       49 VDGNTINLGLWDTAGQEDYNRLRPLSYRGADVFLLAFSLVSKASYENVSKKWVPELRHYA
ATRAC4_ROP2       48 VDGNTVNLGLWDTAGQEDYNRLRPLSYRGADVFILAFSLISKASYENIAKKWIPELRHYA
ATRAC5_ROP4       49 VDGNTVNLGLWDTAGQEDYNRLRPLSYRGADVFILAFSLISKASYENVAKKWIPELRHYA
ATRAC6_ROP5       49 VNGATVNLGLWDTAGQEDYNRLRPLSYRGADVFILAFSLISKASYENVSKKWIPELKHYA
ATRAC7_ROP9       49 VDGQIVNLGLWDTAGQEDYSRLRPLSYRGADIFVLAFSLISKASYENVLKKWMPELRRFA
ATRAC8_ROP10      51 VEGITVNLGLWDTAGQEDYNRLRPLSYRGADVFVLAFSLISRASYENVFKKWIPELQHFA
ATRAC9_ROP8       61 VDGKTVNLGLWDTAGQEDYNRVRPLSYRGADVFILAFSLISRPSFENIAKKWVPELRHYA
ATRAC10_ROP11     51 VEGTTVNLGLWDTAGQEDYNRLRPLSYRGADVFVLSFSLVSRASYENVFKKWIPELQHFA
ATRAC11_ROP1      49 VNGSTVNLGLWDTAGQEDYNRLRPLSYRGADVFILAFSLISKASYENVSKKWIPELKHYA

AtRAC1_ROP3_ROP  109 PGVPIVLVGTKLDLRDDKQFFIDHPGAVPITTAQGEELKKLIGAPAYIECSSKTQENVKG
AtRAC2_ROP3_ROP  109 PGIPIVLVGTKLDLRDDKQFLKDHPGAASITTAQGEELRKMIGAVRYLECSSKTQQNVKA
AtRac3_ROP6      109 PGVPIILVGTKLDLRDDKQFFAEHPGAVPISTAQGEELKKLIGAPAYIECSAKTQQNVKA
ATRAC4_ROP2      108 PGVPIILVGTKLDLRDDKQFFIDHPGAVPITTNQGEELKKLIGSAVYIECSSKTQQNVKA
ATRAC5_ROP4      109 PGVPIILVGTKLDLRDDKQFFIDHPGAVPITTNQGEELKKLIGSPIYIECSSKTQQNVKA
ATRAC6_ROP5      109 PGVPIVLVGTKLDLRDDKQFFIDHPGAVPITTVQGEELKKLIGAPAYIECSSKSQENVKG
ATRAC7_ROP9      109 PNVPIVLVGTKLDLRDDKGYLADHT--NVITSTQGEELRKQIGAAAYIECSSKTQQNVKA
ATRAC8_ROP10     111 PGVPIVLVGTKMDLREDRHYLSDHPGLSPVTTSQGEELRKHIGATYYIECSSKTQQNVKA
ATRAC9_ROP8      121 PTVPIVLVGTKSDLRDNMQFPKNYPGACTIFPEQGQELRKEIGALAYIECSSKAQMNVKA
ATRAC10_ROP11    111 PGVPLVLVGTKLDLREDKHYLADHPGLSPVTTAQGEELRKLIGATYYIECSSKTQQNVKA
ATRAC11_ROP1     109 PGVPIVLVGTKLDLRDDKQFFIDHPGAVPITTAQGEELRKQIGAPTYIECSSKTQENVKA

AtRAC1_ROP3_ROP  169 VFDAAIRVVLQPPK-QKKK-----KSKAQKACSIL-----------
AtRAC2_ROP3_ROP  169 VFDTAIRVALRPPKAKKKI--KPLKTKRSRICFFL-----------
AtRac3_ROP6      169 VFDAAIKVVLQPPKNKKKK-----KRKSQKGCSIL-----------
ATRAC4_ROP2      168 VFDAAIKVVLQPPKQKKKK-----KNKNR--CAFL-----------
ATRAC5_ROP4      169 VFDAAIKVVLQPPKQKKKK-----KNKNR--CVFL-----------
ATRAC6_ROP5      169 VFDAAIRVVLQPPK-QKKK-----KNKAQKACSIL-----------
ATRAC7_ROP9      167 VFDTAIKVVLQPPRRKEVP--RRRKNHRRSGCSIASIVCGGCTAA-
ATRAC8_ROP10     171 VFDAAIKVVIKPAVKQKE---KKKKQKPRSGC-LSNILCGKN----
ATRAC9_ROP8      181 VFDEAIKVVLHPPSKTKKR------KRKIGLCHVL-----------
ATRAC10_ROP11    171 VFDSAIKEVIKPLVKQKEKTKKKKKQKSNHGC-LSNVLCGRIVTRH
ATRAC11_ROP1     169 VFDAAIRVVLQPPK-QKKK-----KSKAQKACSIL-----------
```
