# Supplementary material for: AtRAC7/ROP9 Small GTPase Regulates A. thaliana Immune Systems in Response to B. cinerea Infection
Source: Int J Mol Sci. 2024 Jan 2;25(1):591. doi: 10.3390/ijms25010591 (PMC10779071; doi:10.3390/ijms25010591)
Supplement: Supplementary file 1 [file ijms-25-00591-s001.zip › Figure S2.pdf]

A

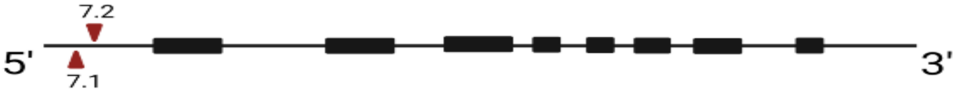

B

TAAATCCAACTTTTAGATAGAAGAGTGGGTTTTTGTAATTTCATTTTGCTAATAAGTTTTTTGAAATTTATTGAGAGGGA
CAATAAAGTTACCCTTTTTAAGACTTTGACTTGAACGAACCTGAAAATCCGACACATTTAACTCTTTCTCTGTCTCGACT
CTCTAGAAAACACACATTTCAATTTTCTTCTCTTTGGTTTTTGAGAAAATATTTCGAGATTTACACTCTTTTTGTTGTCG
CCGCAGTTGATACTCACTGCAAGCGTCTCTTGTAAGCTCATTTAGAGTCTTCTTCACCACTCTTCTTCTTCTTCTTCTTT
AGTTCCTTAAACTTCTGGGTTTTGAGTTTTGTTCTTGGACTAAGAGAATG
