# Supplementary material for: AtRAC7/ROP9 Small GTPase Regulates A. thaliana Immune Systems in Response to B. cinerea Infection
Source: Int J Mol Sci. 2024 Jan 2;25(1):591. doi: 10.3390/ijms25010591 (PMC10779071; doi:10.3390/ijms25010591)
Supplement: Supplementary file 1 [file ijms-25-00591-s001.zip › Table S1.pdf]

	AtRAC9	AtRAC8	AtRAC10	AtRAC7	AtRAC2	AtRAC3	AtRAC4	AtRAC5	AtRAC11	AtRAC6	AtRAC1
AtRAC9	100	66.83	65.33	68.21	73.1	74.62	75.9	75	74.11	74.11	74.11
AtRAC8	66.83	100	90.87	75.86	73.87	74.24	77.95	78.57	77.66	76.65	77.16
AtRAC10	65.33	90.87	100	73.21	73.63	76.26	77.44	78.06	77.16	76.14	77.16
AtRAC7	68.21	75.86	73.21	100	74.37	77.44	77.2	77.84	77.44	76.92	76.92
AtRAC2	73.1	73.87	73.63	74.37	100	81.22	84.1	84.18	84.77	82.74	83.76
AtRAC3	74.62	74.24	76.26	77.44	81.22	100	87.69	89.29	88.32	88.83	89.85
AtRAC4	75.9	77.95	77.44	77.2	84.1	87.69	100	97.44	88.21	88.72	89.23
AtRAC5	75	78.57	78.06	77.84	84.18	89.29	97.44	100	89.8	90.31	90.82
AtRAC11	74.11	77.66	77.16	77.44	84.77	88.32	88.21	89.8	100	95.43	96.95
AtRAC6	74.11	76.65	76.14	76.92	82.74	88.83	88.72	90.31	95.43	100	98.48
AtRAC1	74.11	77.16	77.16	76.92	83.76	89.85	89.23	90.82	96.95	98.48	100
