# Supplementary material for: AtRAC7/ROP9 Small GTPase Regulates A. thaliana Immune Systems in Response to B. cinerea Infection
Source: Int J Mol Sci. 2024 Jan 2;25(1):591. doi: 10.3390/ijms25010591 (PMC10779071; doi:10.3390/ijms25010591)
Supplement: Supplementary file 1 [file ijms-25-00591-s001.zip › Table S4.pdf]

Table S4: List of primers

Name	Sequence (5´-3´)
AtRAC7RTF	AGGATGAAACTCTCCAACTGTGA
AtRAC7RTR	ATCTGCAAATCTATCCACAACCA
Actina F:	GCCATCCAAGCTGTTCTCTC
Actina R:	GCTCGTAGTCAACAGCAACAA
Ubiquitin F:	ATGCAGATCTTCGTGA
Ubiquitin R:	TAGTCAGCCAAGGTCCT
CF150 F:	CCGACAAGGAGAAGCTTAACAAGTT
Cf150 R:	CGGCAGATTTGGATGGACCAGCAAG
CG11	AGCCTTATGTCCCTTCCCTTG
CG12	GAAGAGAAATGGAAAATGGTGAG
