# Supplementary figures and images for: AtRAC7/ROP9 Small GTPase Regulates A. thaliana Immune Systems in Response to B. cinerea Infection
Source: Int J Mol Sci. 2024 Jan 2;25(1):591. doi: 10.3390/ijms25010591 (PMC10779071; doi:10.3390/ijms25010591)

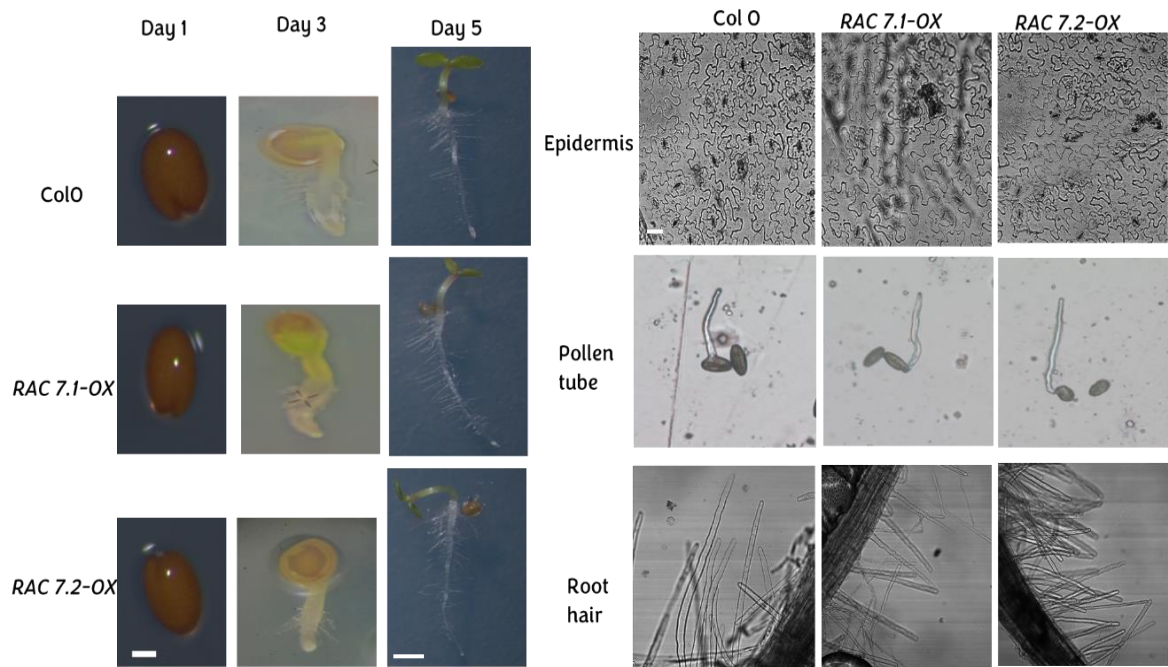

Supplement: Supplementary file 1 [file ijms-25-00591-s001.zip › Figure S3.pdf]

**A**

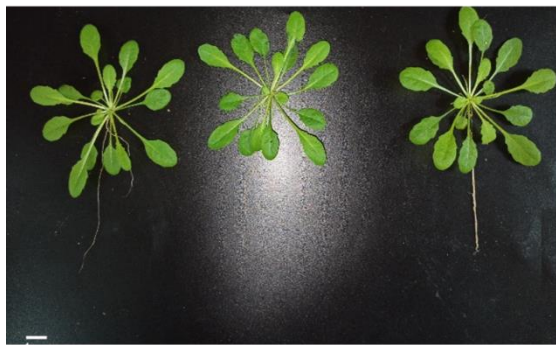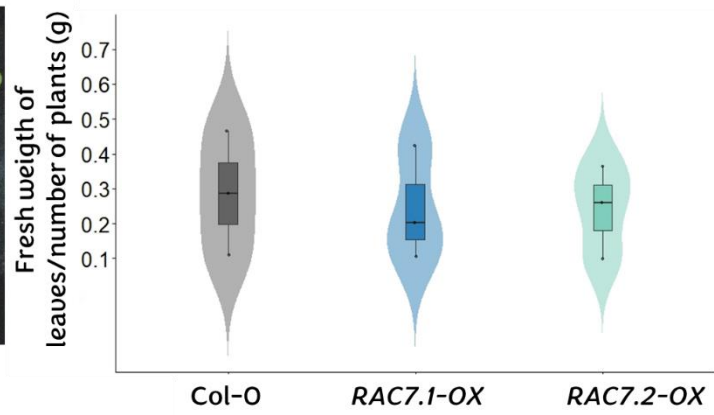

**B**

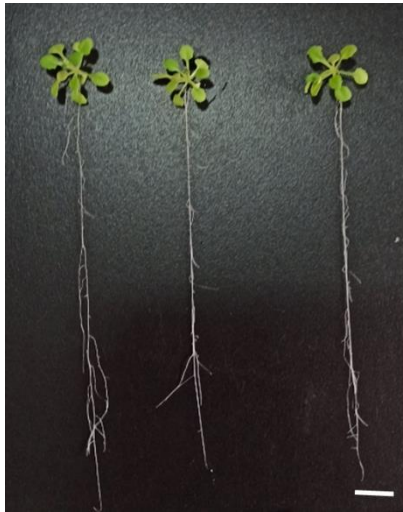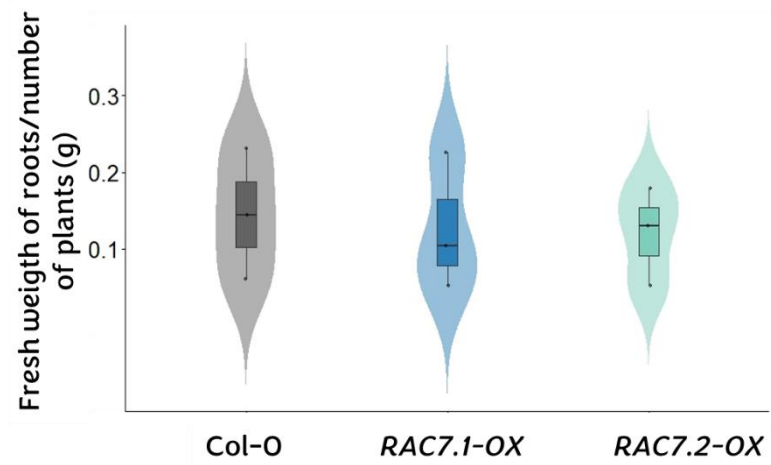

**C**

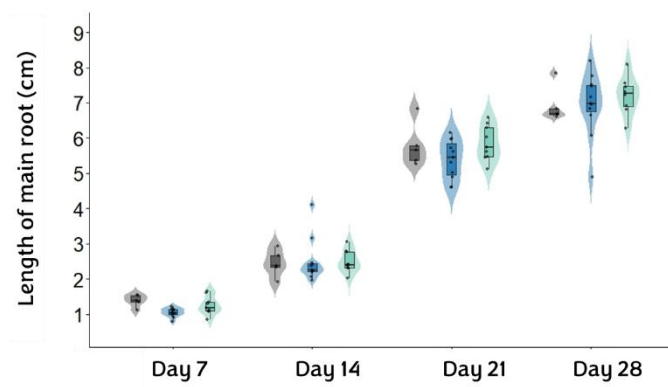

Supplement: Supplementary file 1 [file ijms-25-00591-s001.zip › Figure S4.pdf]

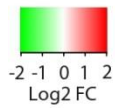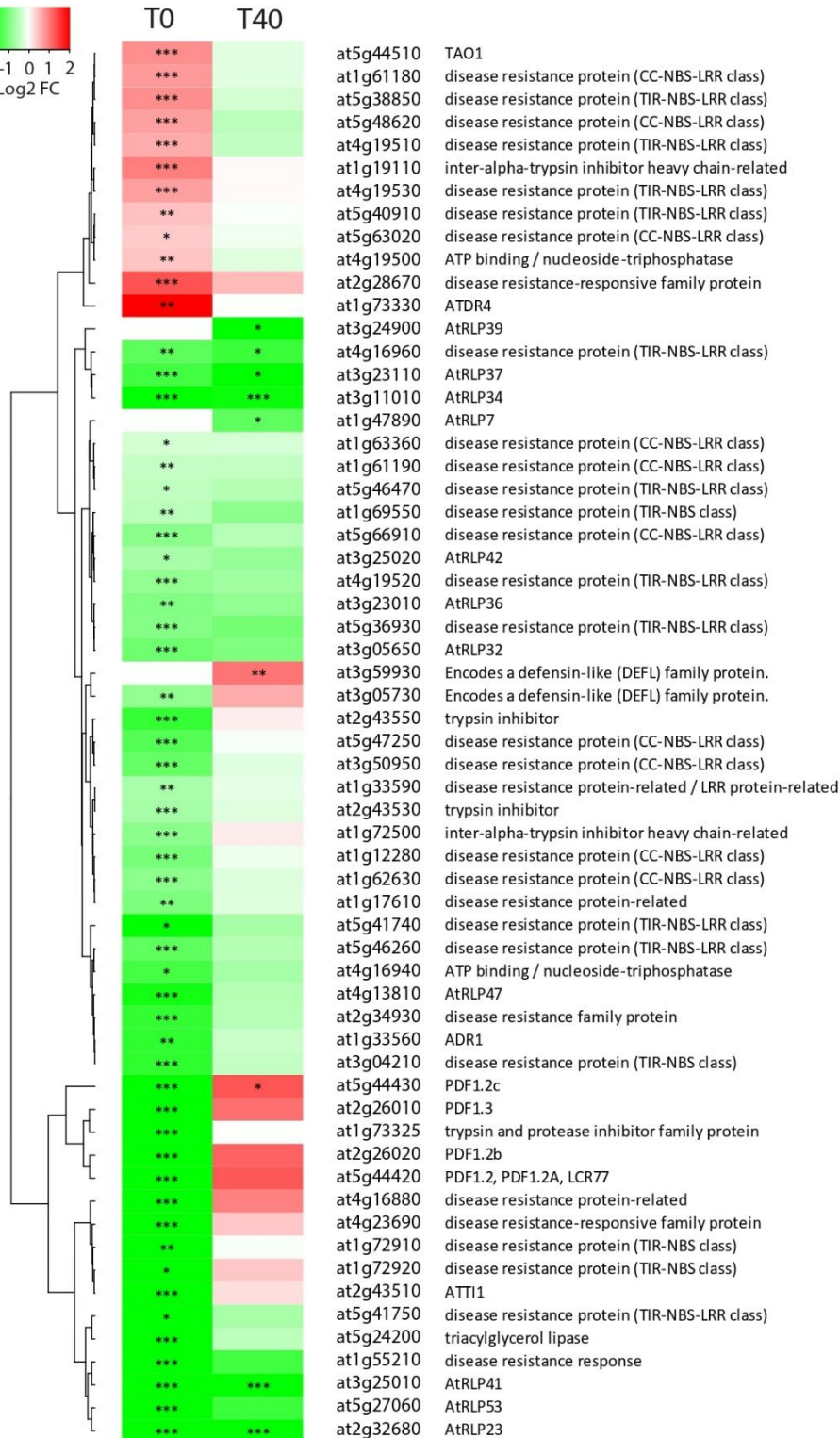

Supplement: Supplementary file 1 [file ijms-25-00591-s001.zip › Figure S5.pdf]

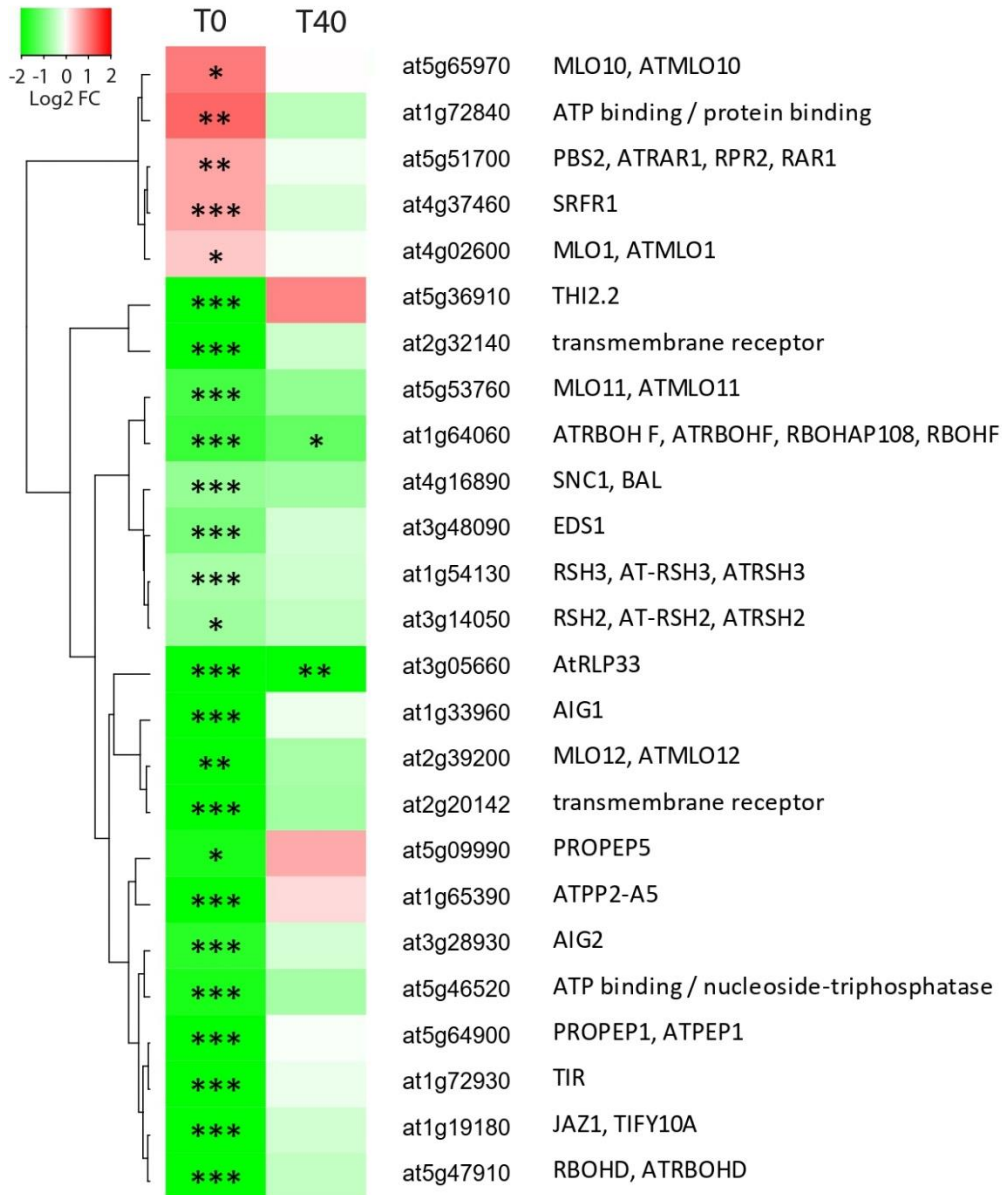

Supplement: Supplementary file 1 [file ijms-25-00591-s001.zip › Figure S6.pdf]

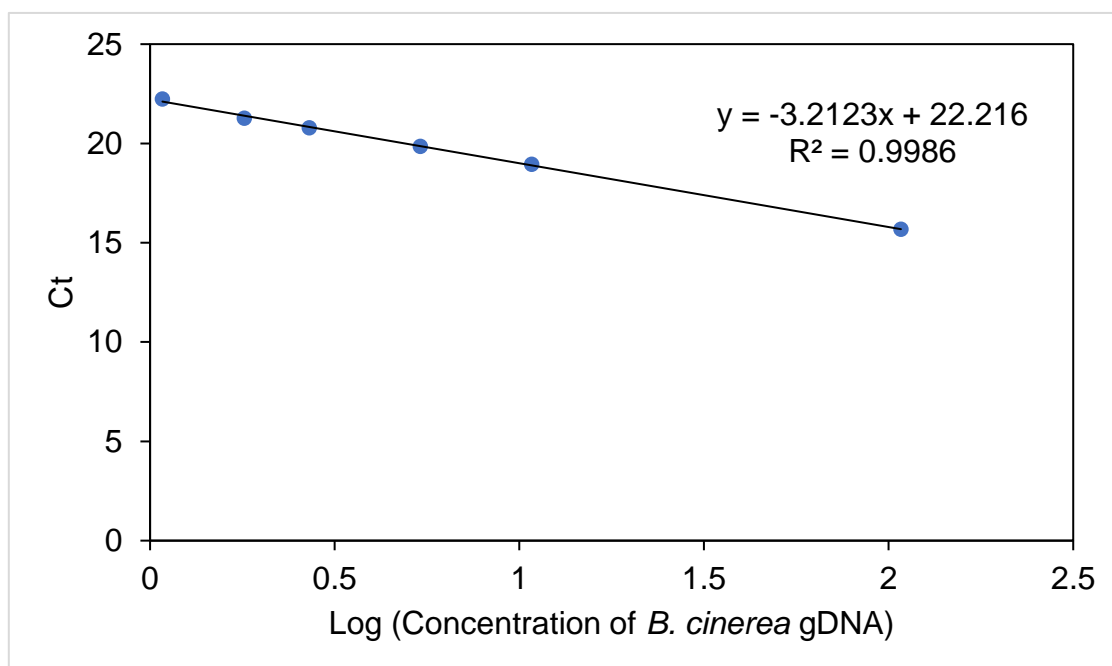

Supplement: Supplementary file 1 [file ijms-25-00591-s001.zip › Figure S7.pdf]
